# Adaptation and validation of the Washington group/unicef child functioning module in a nationally representative sample of Canadian children and youth

**DOI:** 10.1186/s12889-025-23051-1

**Published:** 2025-05-27

**Authors:** Emma Nolan, Katherine Cost, Ryan Miller, Li Wang, Chen Yun-Ju, Jordan Edwards, Eric Duku, Stelios Georgiades, Peter Szatmari, Harriet MacMillian, Charlotte Waddell, Katholiki Georgiades

**Affiliations:** 1 McMaster University, Hamilton, ON Canada; 2 Offord Centre for Child Studies, Hamilton, ON Canada; 3 Queen’s University of Belfast, Northern Ireland, Belfast, UK; 4 Department of Occupational Therapy and Graduate Institute of Behavioral Sciences, College of Medicine, Chang Gung University, Taoyuan, Taiwan; 5 Department of Psychiatry, Chang Gung Memorial Hospital at Linkou, Taoyuan, Taiwan; 6 Department of Psychiatry, The Hospital for Sick Children, Toronto, ON Canada; 7 Department of Psychiatry, Temerty Faculty of Medicine, University of Toronto, Toronto, ON Canada; 8 Centre for Addiction and Mental Health, Toronto, ON Canada; 9 Simon Fraser University BC, Burnaby, Canada

**Keywords:** Children and youth, Functional difficulties, Washington group/Unicef child functioning module, Factor analysis, Latent structure

## Abstract

**Background:**

The Washington Group/UNICEF Child Functioning Module (WG/UNICEF CFM) was developed to identify children and youth with disabilities by assessing functional difficulties. This study focuses on the cognitive, emotional, and behavioral components of the WG/UNICEF CFM, as these domains are particularly relevant to understanding child and youth mental health and developmental functioning. The objective of this study was to examine the latent structure of these domains using a graded response scale in a nationally representative sample of Canadian children and youth aged 5–17 years and to evaluate how this approach captures the dimensional nature of functional difficulties.

**Methods:**

Data for analyses come from the 2019 Canadian Health Survey on Children and Youth (*n* = 33,420). Survey data were collected by Statistics Canada using an electronic questionnaire that was either self-completed online or interviewer-administered by telephone. To assess the latent structure of the WG/UNICEF CFM, analyses were conducted in four linked phases focusing on the following 9 domains: self-care, communication, learning, remembering, concentrating, accepting change, behavior, relationships, and emotions. An exploratory factor analysis (EFA) was conducted first, followed by, confirmatory factor analysis (CFA), then evaluations of measurement invariance across age and sex and external validity using structural equation modeling and instrumental variables.

**Results:**

Results indicated that a two-factor model best described the data, χ2(26, *N* = 16,810) = 619.076, *p* < 0.002, CFI = 0.98, TLI = 0.97, RMSEA = 0.037). Factor one represented Cognitive, Behavioural and Interpersonal Functional Difficulties; while Factor two represented Emotional Functional Difficulties. The construct validity tests supported the distinction between the two factors by demonstrating stronger associations with instrumental variables measuring similar underlying constructs.

**Conclusions:**

This study extends existing research by demonstrating the utility of the WG/UNICEF CFM in assessing cognitive, behavioral, interpersonal, and emotional functional difficulties at the population level in a high-income country. The measure’s strong psychometric properties, ease of use, and cost-free administration support its applicability in general population health surveys of children and youth. Findings highlight the value of a dimensional approach to functional difficulties, offering a more comprehensive understanding of population-level variations in functioning. Integrating this measure into large-scale surveys can facilitate trend monitoring, improve data-driven policy interventions, and support strategic planning for education, healthcare, and social services. These insights contribute to optimizing resource allocation and ensuring equitable access to services that address the diverse functional needs of children and youth.

**Supplementary Information:**

The online version contains supplementary material available at 10.1186/s12889-025-23051-1.

## Background

The term ‘functioning’ has been used to characterize an individual’s ability to meet age and culture-specific role demands across different life domains [1]. Difficulties in meeting these demands exist along a continuum, ranging from no difficulty to severe impairment. However, conceptualizing and measuring child and youth functioning has consistently been challenging. Terms such as “functional impairment” and “disability” are often used interchangeably across disciplines, making it difficult to conceptualize or measure prevalence estimates of either construct. The International Classification of Functioning, Disability and Health (ICF) defines disability based on functional limitations rather than etiology, describing results of interactions between a health condition and contextual factors, as impairments, activity limitations, and participation restrictions [2]. The ICF does not define ‘functional impairment’ as a separate construct but classifies impairments as difficulties in body function or structure that may or may not lead to activity limitations or participation restrictions. Thus, not all impairments result in disability, and not all disabilities stem from a medical diagnosis [2]. A formal diagnosis of disability typically refers to conditions recognized under medical, psychological, or educational classification systems (e.g., DSM-5 for psychiatric disorders, ICD-11 for medical conditions, or special education classifications). Unlike a diagnosed condition, disability in the ICF framework is determined by the extent to which impairments impact daily functioning and social participation.

In the context of cognitive, emotional, and behavioral difficulties, functional impairments may manifest as challenges in self-regulation, social interactions, or adaptive skills, even without a formal diagnosis. In children, this may include attention difficulties, emotional dysregulation, or peer relationship struggles that significantly affect daily life. Thus, treating disability and functional impairment as interchangeable constructs risks overlooking individuals who experience impairments without meeting diagnostic thresholds and vice versa [3, 4]. This distinction is particularly important for mental health conditions, where functional impairment is often a key criterion for diagnosis. However, its measurement remains inconsistent across studies due to variability in how it is operationalized and a lack of standardized assessment tools. The determination of functional impairment is often based on clinical judgment rather than standardized measurement, leading to inconsistencies in how functional limitations are identified and addressed in population-level assessments [4, 5], often lacking consistent operationalization and standardization [4]. This inconsistency presents a major challenge for understanding population-level assessments of youth mental health and well-being. Although several tools measure disability, few are explicitly designed to assess functional difficulties along a continuum, irrespective of health status [6]. As a result, there is limited population-level data on functional difficulties in children and youth [7].

To address this gap, The Washington Group on Disability Statistics and the United Nations Children’s Fund (UNICEF) developed the Washington Group/UNICEF Child Functioning Module (WG/UNICEF CFM) to standardize the measurement of disability and functional difficulties across populations. The module’s purpose is to collect data on child disability and functional difficulties in a standardized and comparable manner globally. It was designed to identify children with disabilities through the assessment of functional difficulties. It is widely used in national surveys and international policy efforts, including Sustainable Development Goal (SDG) data disaggregation [8] to help inform policy on the equalization of opportunities for people with disabilities by improving the quality and international comparability [8]. The WG/UNICEF CFM assesses difficulties in the following functional domains: [9, 10]: vision, hearing, mobility, communication/comprehension, behaviour and learning (for all ages); dexterity and playing (2–4 years); and self-care, communication, learning, remembering, concentrating, accepting change, behaviour, relationships and emotions (5–17 years). To assess the degree of functional difficulties across each of these domains, the following Likert response scales are used: “no difficulty”, “some difficulty”, “a lot of difficulty” and “cannot do at all” or “never”, “a few times a year”, “monthly”, “weekly”, “daily”. Domains can be combined overall, or examined separately, to characterize difficulties within specific domains [10, 11]. Various cut-offs have been recommended for classification purposes. The most inclusive cut-offs recommend classifying presence of a functional difficulty amongst those who endorse ‘Some difficulty’, ‘A lot of difficulty’ or ‘Cannot do at all’ and/or ‘weekly’ or ‘daily’ in at least one domain; whereas the most restrictive cut-offs suggest using ‘Cannot do at all’ and/or ‘daily’. A third approach suggests using ‘A lot of difficulty’ or ‘Cannot do at all’ or ‘Daily’ in at least one domain for classification purposes and has been recommended for studying functional difficulties globally as this approach significantly reduces false positives and variability in prevalence estimates across countries [9]. The WG/UNICEF CFM has been included in the Multiple Indicator Cluster Surveys (MICS) - ongoing household-based surveys of the well-being of children and women in 118 countries - to generate robust country-specific and comparable cross-national data on functional difficulties among children and youth [**8** for a review]. It is estimated that over 70 countries have now included the WG/UNICEF CFM in their national surveys and data from approximately 50 low-to-middle income countries are available [12, *A new way to measure child functioning*, 2021, available at https://data.unicef.org/topic/child-disability/data-collection-tools/module-on-child-functioning/ [accessed 16/12/2023]).

Evaluation of the WG/UNICEF CFM has occurred in several low-to-middle income countries, and results suggest this measure has sound psychometric properties including caregiver/parent- child interrater-reliability [9, 11, 13–15]. In Fiji, the WG/UNICEF CFM was assessed by comparing the diagnostic accuracy of the WG/UNICEF CFM items against standard clinical assessments. Results suggest that the WG/UNICEF CFM had “good” accuracy and overall interrater reliability between parents and teachers (K = 0.68; 95%CI 0.60–0.73; [11]). However, importantly, Sprunt^11^ et al., found that approximately half of the children with moderate impairments and a third of children with severe impairments were not identified using the cut-off ‘a lot of difficulty.’ They found that using ‘cannot do at all’ accurately identified children and youth with severe musculoskeletal impairments but only identified 2% of children and youth with cognitive impairments. In their study, ‘accurate’ identification referred to the instrument’s ability to classify children correctly based on clinical diagnoses or validated measures serving as the benchmark. The authors concluded that these results may be an effect of ambiguity in cut-off levels, suggesting the most restrictive cut-offs may be more accurate in detecting observable musculoskeletal and visual impairments, but may miss a large proportion of children with cognitive difficulties [11]. Another report on the accuracy of these cut-offs in comparison to clinical tests for vision, hearing, musculoskeletal impairment, depression, and history of epilepsy found similar results, whereby using these restrictive cut-offs missed a large proportion of young people with cognitive impairments [16].

Overall, these results suggest that use the use of binary cut-offs, particularly restrictive ones, can overlook the spectrum of functional difficulties by categorizing individuals into dichotomous groups (e.g., with or without difficulty). This approach does not account for varying degrees of difficulty that may exist across different domains, such as mild or moderate difficulties, which can still significantly affect daily life and benefit from intervention. By contrast, a dimensional approach can better acknowledge functional difficulties as existing along a continuum, allowing for more precise characterization and tailored applications, such as identifying individuals for early support or monitoring population-level trends. Indeed, evidence suggests that functioning varies along a continuum requiring graded responses that can be adjusted depending on the outcome being explored [11]. For example, identifying individuals with ‘mild-moderate functional difficulties’ might be important for early intervention, whereas identifying individuals for monetary benefits might require more restrictive cut-offs [17].

While several studies examined the validity and utility of the WG/UNICEF CFM, the data from these studies are all from low-to-middle income countries and use binary classifications of “functional difficulty”. We are unaware of any studies that have examined the latent structure of the WG/UNICEF CFM as a graded tool in a high-income country. There is one study to our knowledge that assessed the latent structure of the WG/UNICEF CFM using total raw scores. It was a study conducted in Uganda [14] and found that a 2-factor structure in children ages 5–17 years best described the data: (1) Motor-Cognition factor (Cronbach’s alpha = 0.90) and (2) Mood factor (Depression & Anxiety; Cronbach’s alpha = 0.90). One limitation of this study is that it combined the domains of physical, learning, remembering and concentrating. In Zia^14^ et al.’s., study, these domains were combined into broader factors based on their factor analysis, which employed graded responses. While this approach was methodologically appropriate, combining domains such as learning, remembering, and concentrating into a single factor may limit the ability to discern the unique contributions of each domain to functional difficulties. This distinction is crucial for understanding domain-specific variations and tailoring interventions accordingly. It is not yet understood; however, how functioning responses may emerge as latent constructs when exploring the data on a domain specific level using graded responses.

Accurately assessing functional difficulties requires measuring behaviors that are not always directly observable, such as cognitive and mood-related challenges, which may form distinct but interrelated constructs. Understanding the latent structure of such measures is essential to ensuring they capture these domains effectively. Although the WG/UNICEF CFM captures a broad range of functional domains, the current study specifically focuses on its cognitive, emotional, and behavioral components, as these are central to understanding mental health and developmental functional difficulties in children and youth. Despite their significance, these domains have been previously underrecognized in population-level assessments using this measure (e.g., Sprunt et al., 2019). The graded response format on the WG/UNICEF CFM provides greater flexibility in establishing thresholds for functional difficulties, allowing for the creation of ordinal scores that reflect a spectrum of severity rather than relying on rigid binary classifications [18]. Examining the latent structure of the WG/UNICEF CFM in a high-income country is particularly important, as the measure has primarily been validated in low- and middle-income contexts. Service systems, diagnostic practices, and societal expectations around functioning can differ substantially in high-income settings, potentially influencing how functional difficulties are reported. Validating the measure in this context enhances confidence in its ability to distinguish cognitive and emotional functional difficulties and supports its use for population-level monitoring, targeted interventions, and cross-national comparisons. A well-defined latent structure also ensures that observed responses align with underlying constructs in a consistent and reproducible manner, thereby improving measurement reliability and the utility of the instrument in diverse contexts [19].

Notably, official descriptions of the WG/UNICEF CFM use the terms “functioning” and “disability” interchangeably, which can obscure important conceptual distinctions, particularly in relation to mental health and cognition. In this study, we use the WG/UNICEF CFM and adopt the ICF Framework to improve conceptual clarity. Throughout this work, we employ the term ‘functional difficulties’ to emphasize the dimensional nature of functioning, rather than a binary classification of disability [2].

This study examines how the conceptual structure of the WG/UNICEF CFM emerges, focusing on functional difficulties related to mental, cognitive, and interpersonal challenges, in a population level context. In addition to assessing its psychometric properties, the findings also highlight how methodological choices, such as scoring approaches and domain aggregation, may influence how functional difficulties are conceptualized and interpreted at the population level. Recognizing these methodological nuances is essential to ensuring valid measurement and cross-national comparability [18, 19].

We emphasize the distinction between “functional difficulties” and “diagnosed disabilities”, as a critical consideration in population-level and mental health research, where traditional diagnostic criteria may fail to capture the full continuum of functional difficulties. By applying a dimensional framework grounded in the ICF and informed by mental health research, this study aims to improve the accuracy of population-level assessments of functional difficulties [2, 5]. Assessing these factors will provide a deeper understanding of variations in functional difficulties across domains, their associated risk factors, and domain-specific patterns over time. Moreover, examining the latent structure of the WG/UNICEF CFM in a high-income, general population sample is essential for global comparisons, particularly given that prior psychometric evaluations have been limited to low-to-middle-income countries [8, 18].

### Objectives

The objective of this study is to examine the latent structure of the cognitive, emotional, and behavioural items of the WG/UNICEF CFM and assess their psychometric properties using a graded response scale in a nationally representative sample of children and youth aged 5–17 years in Canada. This study further explores how adopting a dimensional approach to functional difficulties can enhance the conceptualization of these constructs in population-level research, moving beyond traditional binary classifications. Using factor analysis, this study assesses variations in the latent structure of functional difficulties by age and sex and evaluates the construct validity of the WG/UNICEF CFM [19]. The STROBE reporting guidelines were followed (see Supplement 1, Table 1).

**Table 1:** Response distributions for selected WG/UNICEF CFM items

CFM Items	No difficulty / Never & Few times a year (%)	Some difficulty / Monthly (%)	A lot of difficulty & Cannot do at all / Weekly & Daily (%)
**Self -Care**	96.3	3.1	0.6
**Communication**	96.0	3.0	1.0
**Learning**	83.8	13.4	2.8
**Remembering**	84.4	13.5	2.1
**Concentrating**	92.0	7.0	1.0
**Accepting Change**	71.2	24.2	4.6
**Behaviour**	76.7	20.0	14.5
**Relationships**	82.2	14.5	3.3
**Anxiety**	70.9	11.6	17.5
**Depression**	85.0	8.5	6.5

## Materials and methods

### Study design and participants

Data for analyses come from the 2019 Canadian Health Survey on Children and Youth (CHSCY) [20], a cross-sectional survey that included a nationally representative sample of 47,871 children and youth aged 1–17 years. The Canadian Child Benefit File was used as the sampling frame, with an estimated 98% coverage of the Canadian population aged 1-17years in all provinces and 96% in all territories. Excluded from the survey’s coverage are children and youth living on First Nation reserves and other Indigenous settlements, children and youth living in foster homes and the institutionalized population. The overall survey response rate was 52.1%.

Survey data were collected from February to June 2019 by Statistics Canada using an electronic questionnaire that was either self-completed online or interviewer-administered by telephone. Respondents for the 2019 CHSCY included the person most knowledgeable (PMK) about the selected child or youth (most often the mother), and youth *≥* 12 years (questions relating to suicide were asked of 15–17-year-olds). The sample for analyses for the present study includes children and youth aged 5–17-years (*n* = 33,420). The sample was evenly split between males (50.7%) and females (49.3%), and between age groups (5–8 = 33.9%, 9–12 = 32.7%, 13–17 = 33.4%) with a mean age of 10.6 years. Parental characteristics were also reported, family structure was coded as “one and no biological parents in home” (28.4%) and “two biological parents in home” (71.6%). Parental education was coded as high school and less (0), less than a bachelor’s degree (1) and bachelor’s degree and above (2). For a more detailed breakdown of the sample characteristics please see Table 2 in supplementary materials.

**Table 2:** Fit statistics for exploratory factor analysis of selected items from the WG/UNICEF CFM

Factors	x2	df	*p*	CFI	TLI	RMSEA	BIC
1 Factor	2468.795	35	< 0.001	0.923	0.902	0.064	20902.9
**2 Factor**	**619.076**	**26**	**< 0.002**	**0.981**	**0.968**	**0.037**	**8928.891**
3 Factor	197.041	18	< 0.003	0.994	0.986	0.024	3615.087

Note: Bolded option represents the best fitting / selected factor model

### Child and youth functional difficulty

The caregiver version of the WG/UNICEF CFM was used to assess functional difficulties. Two versions of the WG/UNICEF CFM were administered : 2–4-year-old and 5–17-year-old versions. The present study focused on the 5–17-year-old version (Washington Group / UNICEF Child Functioning Modules).

For the purposes of the present study, 16 items assessing the functional domains of vision, hearing and mobility were dropped from the analyses because the item structure and response options for these domains are distinct from all other domains. Additionally, this decision was further based on conceptual considerations, as these physical and sensory domains are distinct from the psychosocial and developmental aspects that were the primary focus of this study. Including these domains would have introduced additional constructs that require separate validation and potentially distinct measurement approaches. Future research could explore these domains in greater detail to complement the findings of this study.

The WG/UNICEF CFM has 11 items that assess the following functional domains: self -care (1 item), communication (2 items), learning (1 item), remembering (1 item), concentrating (1 item), accepting change (1 item), behaviour (1 item), relationships (1 item) and emotions (2 items anxiety and depression) (see the Washington Group/UNICEF Child Functioning Measure). For the communication items the two questions asked of respondents were (1) When “NAME ”_speaks, do they have difficulty being understood by people inside of this household? and (2) When “NAME” speaks, do they have difficulty being understood by people outside of this household? A sensitivity analysis using these two questions as separate items revealed that the model only had acceptable fit (RSMEA = 0.053, CFI = 0.947 and TLI = 0.932). A modification analyses was run to explore further, and results demonstrated a value below 10, indicating that treating these items as separate does not make significant difference to the explained variance. Therefore, these two items were combined into one variable representing Communication and was coded as (0) no difficulty (selected no to both communications questions), (1) some difficulty (selected at least some difficulty to one of the questions) and (2) a lot of difficulty and cannot do at all (options selected for at least one of the questions). For the final analyses, 10 items were included measuring 9 functional domains. Response options, except for the items relating to emotions, were rated on a Likert scale from 1–4, ‘no difficulty’, ‘some difficulty’, ‘a lot of difficulty’ and ‘cannot do at all’. The final two response options were combined due to low endorsement of the response option ‘cannot do at all’ (approximately 0.30% for the item assessing relationships and between 0.06% − 0.19% across all other domains). The response options were recoded as (0) no difficulty, (1) some difficulty, and (2) a lot of difficulty and cannot do at all. Response options for the items assessing the emotions domain ranged from 1–5, from ‘Daily’, ‘Weekly’, ‘Monthly’ ‘A few times a year’ and ‘Never’ and were recoded and combined as follows: (0) never and a few times a year, (1) monthly, (2)weekly and daily. The items ‘weekly’ and ‘daily’ and ‘Never’ and ‘a few times a year’ were collapsed into one category for two reasons 1) low endorsement (approximately 1% for appearing sad/depressed daily and 5% for appearing anxious/nervous/worried daily), and 2) collapsing these categories ensured consistency across other domains.

### Long term mental health and neurodevelopmental conditions

The PMK was asked if their child had been diagnosed by a health professional with the following conditions expected to last or have lasted longer than six months: learning disability, anxiety disorder, mood disorder, eating disorder, Attentional Deficit Disorder (ADD) or Attentional Deficit Hyperactivity Disorder (ADHD), and autism spectrum disorder (ASD). Mood or anxiety disorders were combined into one variable and coded as (0) neither disorder and (1) at least one disorder. All other diagnoses were treated separately and coded as 0 (no) and 1 (yes).

### Self-perceived health

The PMK was asked “In general how is (Name) Health” and “In general how is (Name) mental health?’. Responses were on a 5-point Likert scale from ‘excellent’, ‘very good’, ‘good’, ‘fair’ ‘poor’ and recoded to ‘0 = ‘excellent, very good or good’ and ‘1 = ‘Fair or Poor’. These questions are widely used in general health surveys and have demonstrated adequate reliability and validity [21, 22].

### Special education needs

The PMK was asked whether their child had an individual education plan (IEP), Special Education Plan (SEP) or Inclusion and Intervention Plan (IIP). In Canada, Special Education Needs plans, IEPs, SEPs, and IIPs are formalized documents outlining accommodations and supports for students with identified needs. These plans are typically provided to children with learning disabilities, developmental disabilities, behavioral challenges, or physical disabilities. Eligibility is determined through a collaborative process involving teachers, school administrators, and parents, often informed by assessments conducted by educational psychologists or healthcare professionals. The goal is to ensure equitable access to education tailored to the student’s unique requirements. If the PMK indicated that their child had either an IEP, SEP or IIP, they were asked to indicate which of the following learning exceptionalities or special education needs their plan was associated with: ‘a permanent physical disability’ (0 = no, 1 = yes) and ‘cognitive, behavioral or emotional disability’ (0 = no, 1 = yes).

### Suicidality

These questions were asked directly to youth aged 15–17-years (*n* = 6,915). Three questions were asked; “During the past 12 months, did you ever feel so sad or hopeless almost every day for two weeks or more in a row that you stopped doing some usual activities”; “In the past 12 months, did you ever seriously consider attempting suicide or taking your own life”; “Have you ever attempted suicide or tried taking your own life”. The response to these three questions were coded as “no = 0” and “yes = 1”. Each of these items were used to create a latent variable indicative of the presence of suicidality.

### Health services

The PMK was asked whether their child required or received services for each of the following concerns (0 = no, 1 = yes): speech or language difficulties, difficulties with mobility/dexterity, difficulties focusing or controlling behaviour, mental health issues and learning difficulties. While these questions were framed under the broader context of accessing health care in the survey, some of these services (e.g., speech or language, learning) may not traditionally be categorized as ‘health services’ in other contexts. To ensure clarity, we grouped these services together to capture the broad spectrum of support needs identified in the survey. Responses to these questions were used to construct two variables. The first was a latent variable indicative of requiring or receiving health services for difficulties in speech or language, mobility/dexterity, focusing or controlling behaviour and learning difficulty. The second represented an observed variable indicative of requiring or receiving care for mental health difficulties (0 = no, 1 = yes).

### Sex assigned at birth

Sex assigned at birth was assessed by asking the PMK “What was (NAME’s) sex at birth? coded as (0) Male, and (1) Female.

### Analysis

The statistical software Mplus 7.0 [23] was used to conduct the analysis in four, linked phases.

### Hypotheses

Guided by prior research and theoretical considerations, we expected that the WG/UNICEF CFM items related to cognitive, emotional, and behavioural functioning would form distinct latent factors, though the exact structure was examined empirically. Specifically, we anticipated that the items would cluster into factors representing (1) Cognitive, Behavioural, and Social Functional Difficulties, which may include self-care, communication, learning, remembering, concentrating, accepting change, behaviour, and relationships, and (2) Emotional Functional Difficulties, capturing emotional regulation and mood-related challenges (anxiety and depression). Exploratory Factor Analysis (EFA) was conducted to identify the underlying factor structure without imposing a predetermined model, followed by Confirmatory Factor Analysis (CFA) to evaluate model fit in an independent sample. Measurement invariance testing was conducted to assess the stability of the identified structure across age and sex.

### Analytical phases 1–3

In phase 1, a random sample of 50% of respondents was selected. An exploratory factor analysis (EFA), using oblique promax rotation, was conducted on all selected items from the WG/UNICEF CFM to determine the underlying factor structure of these specific domains and assess the associations of the items with their hypothesized subscales, which represent the latent constructs measured by the instrument (i.e. internal factor structure). As latent indicators were categorical, we used mean, and variance adjusted diagonally weighted least squares (WLSMV) estimation with delta parameterization.

In phase 2, the other 50% of the sample was used to conduct a confirmatory factor analysis (CFA), to assess the fit of the structure in an independent sample. CFA results were evaluated using four model fit criteria: (1) A non-significant Chi-Square; (2) Root Mean Square Error of Approximation (RMSEA, < 0.05); (3) Comparative Fit Indices (CFI, > 0.95); and (4) Weighted Root Mean Square Residual (WRMR, < 1.00).

In phase 3, we examined measurement invariance across age groups (5–8 years of age, 9–12 years of age, 13–17 years of age) and sex (male, female). Configural invariance (the same items were associated with the same factors across all groups), metric invariance (factor loadings were similar across groups), and scalar invariance (factor means are equivalent across groups) [24] were evaluated using model fit indices. We required that two out of three model fit criteria (CFI > 0.95, RMSEA < 0.08, WRMR < 0.08) AND two of three change in model fit criteria (CFI >-0.02 and − 0.01, RMSEA < 0.03 and 0.01, and WRMR < 0.03 and 0.01 for metric and scalar respectively) were met to have confidence in measurement invariance [25, 26]. Internal convergent validity assesses the items that make up a subscale and compares the shared variance with that subscale, in relation to measurement error, which is assessed using the Average Variance Extracted (AVE). In assessing AVE, a value of AVE ≥ 0.5, indicates that at least 50% of the total variance in the items is explained by the scale [27]. Internal discriminant validity is assessed by comparing the shared variance within each subscale to the shared variance between subscales and is demonstrated when the square root of AVE, for a given subscale, is larger than the correlations between this subscale and all others [28].

### Analytical phase 4

In phase 4, to evaluate construct validity, we tested the associations between the identified latent factors of the WG/UNICEF CFM and external variables that theoretically align with functional difficulties. We assessed the construct validity by examining and comparing associations between each WG/UNICEGF CFM latent factor and the following instrumental variables: long term health conditions diagnosed by a health professional (anxiety or mood disorder, learning disability, eating disorder, ADHD, and Autism), perceived general health, perceived mental health, special education needs, suicidality, and health services.

Specifically, we hypothesized the Cognitive, Behavioral, and Interpersonal Functional Difficulties factor would be more strongly associated with neurodevelopmental conditions (e.g., ADHD, learning disabilities), special education needs, and perceived learning difficulties, reflecting challenges in executive functioning, social skills, and behavioral adaptation. The Emotional Functional Difficulties factor would show stronger associations with mood/anxiety disorders, suicidality, and perceived mental health, aligning with emotional regulation and internalizing difficulties. Both factors would relate to health service use, with CBIFD more linked to education-related services and EFD to mental health services. We expected both factors to correlate with poorer self-perceived general health, though with varying magnitudes.

We used structural equation models (SEMs) to estimate and compare the strength of associations between each WG/UNICEF CFM latent factor and each instrumental variable. This analysis regresses the latent WG/UNICEF CFM factors on each instrumental variable. In some cases, the instrumental variable was observed and in others it was a latent variable (see Fig. 1 in supplement “Structural equation model for external validity using diagnosed Long-Term Health Condition of anxiety and mood disorders”, for an example). Adequate model fit was defined as CFI ≥ 0.95) and RMSEA ≤ 0.05 [29]. Standardized beta coefficients (β) associated with each of the WG/UNICEF CFM latent factors ensure comparisons are made on a commensurate scale. Differences in the strength of associations between each WG/UNICEF CFM latent factor and instrumental variables were examined using Wald Chi-Square tests.

There was missing data for 185 participants across all variables, and they were excluded from the analyses (final *n* = 33,235). For the WG/UNICEF CFM responses, 2.5% were missing responses on only 1 item, 0.2% on two items, and 0.8% on three or more items. Full information maximum likelihood (FIML) was used to estimate model parameters. Sampling weights were applied in all analyses. It is important to note that each domain within the WG/UNICEF CFM is measured by a single item. While this limits the ability to assess internal consistency, the use of factor analysis and structural equation modeling allows for the evaluation of construct validity by examining the relationships between latent factors and external criteria. This approach helps to mitigate the constraints of single-item measures by leveraging external validation methods.

## Results

Descriptive statistics for selected items used in the present study from the WG/UNICEF CFM are reported in Table 1. The majority of the sample (*> 70%)* endorsed no difficulties for each functional domain included in the present study. The functional domains with the highest frequency of endorsement of difficulties were the behavioral and emotional domains, with 14.5% of the sample reporting a lot difficulty/cannot do at all for controlling behaviour and 17.5% reporting feelings of anxiety, nervousness or worry on a weekly or daily basis.

Overall, the results suggest that functional difficulties as measured by the selected items from the WG/UNICEF CFM is best described as a two-factor construct. Based on the fit statistics for 1, 2, and 3 factor models in the EFA (Table 2), a two-factor model was the best fit on all fit statistics except the chi-square. While the chi-square was significant, this should not lead to rejection of the 2-factor model as the power of the chi-square is positively associated with the sample size [30]. The inspection of the eigenvalues and scree plot also supported a two-factor model, with the third factor having an eigenvalue of 0.27.

The rotated factor loadings of the 2-factor EFA are presented in Table 3 of the supplementary materials. All items load strongly on their respective factors. Factor 1 included items relating to self-care, communication, learning, remembering, concentrating, accepting change, behavior and relationships. Factor 2 included both items from the emotional domain (i.e., depression and anxiety). The standardised factor loadings were positive, high, and statistically significant. Internal consistency was excellent for Factor 1 (Cronbach’s alpha = 0.93) and good for Factor 2 (Cronbach’s alpha = 0.82) [18, 31]. Average internal consistency for the full scale was excellent (Cronbach’s alpha = 0.91).

**Table 3:** Fit indices for confirmatory factor analyses and measurement invariance by age and sex

	χ2	df	*p*-value	RMSEA	WRMR	CFI	TLI	AVE (√AVE)	Intra-factor Correlations
**CFA (2-factor)**									
All (5–17)	-	-	-	0.043	4.590	0.966	0.955	0.76 (0.87)	0.66
**Age Group**									
5–8	-	-	-	0.042	2.670	0.961	0.948	0.69 (0.83)	0.64
9–12	-	-	-	0.043	2.728	0.968	0.957	0.77 (0.88)	0.68
13–17	-	-	-	0.042	2.714	0.972	0.963	0.82 (0.90)	0.68
**Sex**									
Male	-	-	-	0.044	3.405	0.970	0.960	0.76 (0.87)	0.70
Female	-	-	-	0.041	3.195	0.958	0.945	0.76 (0.87)	0.65
**Invariance Tests**									
**Age**									
Configural	2076.641	102	*p* < 0.001	0.042	4.689	0.967	-	-	-
Metric	2138.86	118	*p* < 0.001	0.039	5.163	0.966	-	**-**	**-**
Scalar	1994.924	134	*p* < 0.001	0.035	5.242	0.969	-	-	-
	***Δχ2***	***Δdf***	**p-value**	**ΔRMSEA**	**ΔWRMR**	**ΔCFI**	**-**	-	-
Configural vs. Metric	250.749	16	*p* < 0.001	-0.003	0.474	0.001	-	-	-
Configural vs. Scalar	288.339	32	*p* < 0.001	-0.007	0.553	-0.002	-	**-**	**-**
Metric vs. Scalar	50.082	16	*p* < 0.001	-0.004	0.079	0.003	-	-	-
**Sex**									-
Configural	2148.337	68	*p* < 0.001	0.043	4.669	0.965	-		-
Metric	2140.905	76	*p* < 0.001	0.04	4.908	0.965	-	-	-
Scalar	2056.746	84	*p* < 0.001	0.037	4.983	0.967	-	-	-
	***Δχ2***	***Δdf***	**p-value**	**ΔRMSEA**	**ΔWRMR**	**ΔCFI**		-	-
Configural vs. Metric	131.772	8	*p* < 0.001	-0.003	0.239	0.000	-	-	-
Configural vs. Scalar	175.064	16	*p* < 0.001	-0.006	0.314	0.002	-	-	-
Metric vs. Scalar	49.028	8	*p* < 0.001	-0.003	0.075	0.002	-	-	-

Table 3 shows the results of the confirmatory factor analyses (CFA) and measurement invariance. The CFA and measurement invariance were conducted on the second half of the sample (*n* = 16,810) and further supported the 2-factor model. The two-factor model had excellent model fit according to our criteria. The configural, metric, and scalar models of invariance also indicted adequate model fit based on the differences in the CFI, RMSEA and WRMR for age and sex for the WG/UNICEF CFM (Table 3). Internal convergent and discriminant validity were achieved across all models as AVEs were greater than 0·5 and all square roots of the AVEs were greater than any inter-factor correlations. Table 4 shows the rotated factor loadings of the CFA for the selected items from the WG/UNICEF CFM. The standardised factor loadings were positive, high, and statistically significant, and supported the EFA results.

**Table 4:** Rotated factor loadings from confirmatory factor analysis model of selected items from the WG/UNICEF CFM

WG/UNICEF CFM items	Factor 1	Factor 2
All:		
Selfcare	0.764	
Communication	0.734	
Learning	0.845	
Remembering	0.809	
Concentrating	0.750	
Accepting Change	0.768	
Behaviour	0.768	
Relationships	0.725	
Anxiety		0.910
Depression		0.836

**Table 5:** Associations between instrumental variables and latent factors of the WG/UNICEF CFM

Variable	Factor 1 Cognitive, Behavioural and Interpersonal Difficulties		Factor 2 Emotional Functional Difficulties		β Differences		Model Fit Statistics		
	**β**	**SE**	**β**	**SE**	∆ (β_1_–β_2_)	**SE**	**RMSEA**	**CFI**	**TLI**
**Long Term Health Condition (Observed Variables)**									
Anxiety or Mood	0.264***	0.009	0.383***	0.008	-0.119***	0.009	0.040	0.962	0.950
Learning Disability	0.387***	0.008	0.215***	0.009	0.172***	0.009	0.042	0.957	0.944
Eating disorder	0.071***	0.012	0.097***	0.011	-0.026***	0.012	0.040	0.961	0.948
AD/HD	0.385***	0.009	0.239***	0.010	0.146***	0.009	0.040	0.962	0.950
Autism	0.279***	0.010	0.164***	0.009	0.115***	0.010	0.040	0.957	0.944
**Self-rated health (Observed Variables)**									
General Health	0.138***	0.009	0.136***	0.009	0.002	0.01	0.04	0.966	0.955
Mental Health	0.314***	0.008	0.390***	0.008	-0.076***	0.01	0.04	0.96	0.948
**School Special Education (Latent Variables)**							0.027	0.968	0.962
Physical Disability	0.159***	0.011	0.079***	0.01	0.080***	0.01			
Cognitive/ behavioral/ emotional disability	0.457***	0.008	0.262***	0.01	0.194***	0.01			
**Suicidality (15–17) (Latent Variables)**									
Distress/Hopelessness/Suicidal ideation / Suicidal attempt /	0.371***	0.026	0.581***	0.023	-0.210***	0.03	0.04	0.969	0.961
**Accessing health care for**:									
Speech, language, mobility controlling behaviour, learning difficulties (latent)	0.947***	0.008	0.554***	0.015	0.393***	0.015	0.04	0.951	0.94
Mental Health care (observed)	0.285***	0.008	0.415***	0.008	-0.130***	0.009	0.04	0.962	0.95

Latent = observed variables that were combined to create a latent variable. The strongest and most significant associations with the respective factors are indicated by bolded β values for both observed and latent variables

Table 5 shows associations between instrumental variables and latent factors were assessed using a series of structural equation models (SEMs, see Fig. 1 “Structural equation model for external validity using diagnosed Long-Term Health Condition of anxiety and mood disorders”, in supplement). All models met adequate model fit criteria. There was strong and consistent evidence for convergent and discriminant validity across all instrumental variables examined. For example, when comparing differences in regression coefficients (β1–β2) of Factor 1 and Factor 2, Factor 2 showed a stronger association with the anxiety and mood variables (β difference = -0.119, p < 0.001); while a diagnosis of a learning disability was more strongly associated with Factor 1 (β difference = 0.175, p < 0.001).

## Discussion

This study focused on assessing cognitive, behavioural, interpersonal and emotional functional difficulties at a population-level in children and youth. This work used the WG/UNICEF CFM, to build on the ICF Framework, and addresses limitations in previous population-level research, where functional difficulties are often equated to diagnostic labels. Previous approaches may fail to capture the variability in cognitive, emotional, and social functioning, limiting the ability to identify nuanced patterns of potential need. Our findings supported our hypotheses and highlights the importance of using a dimensional approach when assessing these functional difficulties in children and youth. This study highlights how methodological choices, such as the selection and grouping of functional domains, might influence the conceptualization of functional difficulties and the interpretation of latent structures. Given that the WG/UNICEF CFM is widely used for global disability disaggregation, understanding these measurement considerations is essential for ensuring valid and comparable estimates across diverse populations and research contexts.

We evaluated the psychometric properties of specific domains of the WG/UNICEF CFM in a nationally representative sample of Canadian children and youth, aged 5–17-year-old. Extending previous psychometric evaluations, which have focused on low-to-middle-income countries, our study assessed this measure in a high-income country, extending its applicability. Findings suggest that the WG/UNICEF CFM is a valid tool for assessing functional difficulties in high-income settings.

Evidence from this study supports a two-factor model of functional difficulties, where Factor 1 represents Cognitive, Behavioural, and Interpersonal Functional Difficulties (CBIFD), and Factor 2 represents Emotional Functional Difficulties (EFD). With our adaptation of the WG/UNICEF CFM, excluding the functional domains of vision, hearing, and mobility, our factorial validity work remains consistent with previous psychometric research (Zia et al., 2019), however extends these findings by incorporating a graded response approach to these domains. The graded response approach has previously been suggested for population-based analyses of functional difficulties [14] but has not yet been tested. Previous conceptualizations, which have relied on binary classifications, may obscure meaningful variation in functional difficulties by forcing individuals into rigid categories that do not fully capture the spectrum of functional experiences. In contrast, a graded response approach allows for a more nuanced measurement of functional difficulties, preserving individual differences in severity and identifying subpopulations that may otherwise be overlooked. This approach enhances the precision of population-level assessments by more accurately reflecting the distribution and variability of functional challenges across individuals, and capturing a wider continuum of functional difficulties.

Confirmatory factor analysis and measurement invariance testing provided additional support for the two-factor structure, indicating that it was stable across age and sex. The construct validity of the two-factor model was further demonstrated through associations with external variables. CBIFD was more strongly linked to neurodevelopmental conditions such as ADHD and learning disabilities, while EFD showed stronger associations with mood and anxiety disorders, suicidality, and perceived mental health (Table 5). These differential associations support the conceptual distinction between cognitive/behavioural and emotional functional difficulties, reinforcing the validity of the two-factor structure.

Historically, measuring change in mental health symptoms has been a core outcome of interest in both observational and clinical research studies. However, recent research has demonstrated that improvement in functioning now represents a concept of high priority for young people who are receiving mental health care [32]. This study provides initial support for the WG/UNICEF CFM as a reliable tool for assessing functional difficulties, and improvements, across distinct domains, allowing for more precise identification of differences in correlates and outcomes. We argue that quantifying functional difficulties along a continuum provides a more nuanced understanding of general population needs. This approach facilitates the identification of subgroups within a population who may be experiencing mild-to-moderate difficulties, and can therefore enable early interventions that may prevent escalation into more severe challenges. Additionally, capturing the spectrum of functional difficulties allows for more precise monitoring of health trends and tailoring of policy responses to address the unique needs of diverse demographic groups. Such granularity is essential for effective resource allocation and addressing disparities in access to support services [17].

### Strengths & limitations

This study has some key strengths. It is the first study to our knowledge to explore the underlying latent structure of functional difficulties using the WG/UNICEF CFM in a high-income country using graded responses of levels of functional difficulty. The sample used is a large, nationally representative sample of Canadian children and youth aged 5–17 years. Additionally, this study employs a comprehensive and methodologically rigorous analytical framework, incorporating Exploratory Factor Analysis, Confirmatory Factor Analysis, and measurement invariance techniques alongside validation procedures. Leveraging a large sample, which was bifurcated for analytical purposes, this approach facilitates a thorough examination of the psychometric properties of WG/UNICEF CFM.

There are some noteworthy limitations, including the need to extend our psychometric evaluation to more ethnically diverse populations to examine the extent to which cultural differences may influence the underling factor structure. It is important to note, however, that the WG/UNICEF CFM has been widely used in many low-middle income countries through the Multiple Indicators Cluster Surveys, and all psychometric evaluations of the instrument have used samples from these counties [see 8 for a review of these]. Further, in the present study, we dropped items relating to hearing, vision, and mobility functional difficulties, therefore we have not assessed these domains as they relate to latent structure and dimensional scoring. These domains were excluded from the adaptation and analysis due to conceptual considerations about the scope of functioning assessed. Specifically, this study aimed to explore domains that align more closely with psychosocial and developmental functioning, rather than physical or sensory impairments. Future research could address these domains separately to ensure a comprehensive understanding of functional difficulties across all aspects of child and youth functioning. Additionally, it is important to acknowledge that each domain of functioning in the WG/UNICEF CFM is assessed using a single-item measure. While this allows for efficient assessment in large-scale surveys, it may limit measurement precision and reliability. Single-item indicators do not capture the full breadth of a construct and may be more susceptible to measurement error compared to multi-item scales. Future research should consider whether additional items per domain would enhance construct validity and measurement reliability, particularly for cognitive, emotional, and behavioral functioning.

This study focused on functional difficulties, which assess the degree of difficulty in daily activities and may occur with or without a diagnosis. Preliminary analyses showed high correlations between functional difficulties and diagnoses, which does support the use of functional measures in this context, however future research will explore their interplay in more depth. Lastly, the categorization of family structure as ‘two biological parents in home’ excluded adoptive parents and step parents, which may limit the inclusivity of this measure and the generalizability of findings related to family composition. Nonetheless, this study has demonstrated that the WG/UNICEF CFM tool can be used to examine functional difficulties as graded responses in a high-income country general population sample of children and youth. Assessing functional difficulties as a dimensional continuum will aid in a better understanding of differences and variations that may exist at varying levels in the population. It can also have practical implications for intervention planning and monitoring.

## Conclusion

The results of this study extends the extant literature to demonstrate the utility of the WG/UNICEF CFM in assessing, cognitive, behavioural, interpersonal and emotional functional difficulties at the population level. The psychometric properties, including demonstrated reliability and validity, in combination with its ease of use and no cost, highlights the applicability of the WG/UNICEF CFM as a useful measure for assessing functional difficulties in a general population-based sample of children and youth. The results show that the measure can be reliably adapted to represent two distinct factors: Cognitive, Behavioral, and Interpersonal Functional Difficulties, and Emotional Functional Difficulties. Additionally, the findings support the use of a dimensional approach to understanding functional difficulties, providing a more comprehensive view of population-level variations in functioning. Enabling the assessment of functional difficulties across a broad spectrum, can help support policy development, resource allocation, and service planning at the national and international levels. Specifically, the inclusion of his measure in population-based health surveys, might help identify trends in functional difficulties, monitor disparities in access to services, and inform data-driven policy interventions. The ability to track functional difficulties over time can support governments and organizations in tailoring education, healthcare, and social service frameworks to better address emerging needs within the population. Further, we suggest that by integrating a graded response approach into large-scale assessments, helps refine eligibility criteria for services, optimize support provision, and enhance long-term planning for disability and mental health programs.

The WG/UNICEF CFM presents a promising tool for population-level surveillance of functional difficulties in children and youth, particularly in high-income countries where comprehensive, comparable data on child functioning remain limited. Future research should continue to explore its adaptability across diverse populations and examine how functional difficulties interact with broader social determinants of health to inform more effective, equitable policy and service delivery.

## Electronic supplementary material

Below is the link to the electronic supplementary material.

Supplementary Material 1

## Data Availability

The data that support the findings of this study are available from [Statistics Canada] but restrictions apply to the availability of these data, which were used under license for the current study, and so are not publicly available. Data are however available with access and with permission of Research Data Centres data repositories and Statistics Canada.

## Declarations

All manuscripts must contain the following sections under the heading.
