# Supplementary material for: Adaptation and validation of the Washington group/unicef child functioning module in a nationally representative sample of Canadian children and youth
Source: BMC Public Health. 2025 May 27;25:1954. doi: 10.1186/s12889-025-23051-1 (PMC12107942; doi:10.1186/s12889-025-23051-1)
Supplement: Supplementary file 1 — Supplementary Material 1 [file 12889_2025_23051_MOESM1_ESM.docx]

**Supplement 1:**

**Table 1:** STROBE Guidelines.

|  | **Item No.** | **STROBE items** | **Location in manuscript where items are reported** |
| --- | --- | --- | --- |
| **Title and abstract** | | | |
|  | 1 | (a) Indicate the study’s design with a commonly used term in the title or the abstract (b) Provide in the abstract an informative and balanced summary of what was done and what was found | Pages 1 and 2 |
| **Introduction** | | | |
| Background rationale | 2 | Explain the scientific background and rationale for the investigation being reported | Pages 3 - 7 |
| Objectives | 3 | State specific objectives, including any prespecified hypotheses | Page 7 |
| **Methods** | | | |
| Study Design | 4 | Present key elements of study design early in the paper | Page 7. Key methods can be found at the end of the background section. |
| Setting | 5 | Describe the setting, locations, and relevant dates, including periods of recruitment, exposure, follow-up, and data collection | Page 8 |
| Participants | 6 | *(a) Cohort study* - Give the eligibility criteria, and the sources and methods of selection of participants. Describe methods of follow-up  *Case-control study* - Give the eligibility criteria, and the sources and methods of case ascertainment and control selection. Give the rationale for the choice of cases and controls  *Cross-sectional study* - Give the eligibility criteria, and the sources and methods of selection of participants  *(b) Cohort study* - For matched studies, give matching criteria and number of exposed and unexposed  *Case-control study* - For matched studies, give matching criteria and the number of controls per case | a) Pages 8 and 9 |
| Variables | 7 | Clearly define all outcomes, exposures, predictors, potential confounders, and effect modifiers. Give diagnostic criteria, if applicable. | Pages 9 - 12 |
| Data sources/ measurement | 8 | For each variable of interest, give sources of data and details of methods of assessment (measurement).  Describe comparability of assessment methods if there is more than one group | Pages 9-12 and Supplement 1 |
| Bias | 9 | Describe any efforts to address potential sources of bias | Page 12-14 |
| Study size | 10 | Explain how the study size was arrived at | Page 12 |
| Quantitative variables | 11 | Explain how quantitative variables were handled in the analyses. If applicable, describe which groupings were chosen, and why | Pages 9-12 |
| Statistical methods | 12 | (a) Describe all statistical methods, including those used to control for confounding  (b) Describe any methods used to examine subgroups and interactions  (c) Explain how missing data were addressed  (d) *Cohort study* - If applicable, explain how loss to follow-up was addressed  *Case-control study* - If applicable, explain how matching of cases and controls was addressed  *Cross-sectional study* - If applicable, describe analytical methods taking account of sampling strategy  (e) Describe any sensitivity analyses | Pages 12-14 |
| Data access and cleaning methods |  | .. |  |
| Linkage |  | .. |  |
| **Results** | | | |
| Participants | 13 | (a) Report the numbers of individuals at each stage of the study (*e.g.*, numbers potentially eligible, examined for eligibility, confirmed eligible, included in the study, completing follow-up, and analysed)  (b) Give reasons for non-participation at each stage.  (c) Consider use of a flow diagram | Page 12-15 + Supplement 1 |
| Descriptive data | 14 | (a) Give characteristics of study participants (*e.g.*, demographic, clinical, social) and information on exposures and potential confounders  (b) Indicate the number of participants with missing data for each variable of interest  (c) *Cohort study* - summarise follow-up time (*e.g.*, average and total amount) | Table 1, 2 & Page 12 & Table 2 in supplement 1.  b) We had minimal missing data, discussed page 13.  c) Not applicable |
| Outcome data | 15 | *Cohort study* - Report numbers of outcome events or summary measures over time  *Case-control study* - Report numbers in each exposure category, or summary measures of exposure  *Cross-sectional study* - Report numbers of outcome events or summary measures | Page 15  Tables 1 & 2 |
| Main results | 16 | (a) Give unadjusted estimates and, if applicable, confounder-adjusted estimates and their precision (e.g., 95% confidence interval). Make clear which confounders were adjusted for and why they were included  (b) Report category boundaries when continuous variables were categorized  (c) If relevant, consider translating estimates of relative risk into absolute risk for a meaningful time period | a) Pages 12-15  b) Pages 9-12  c) N/A |
| Other analyses | 17 | Report other analyses done—e.g., analyses of subgroups and interactions, and sensitivity analyses | Pages 12-14 |
| **Discussion** | | | |
| Key results | 18 | Summarise key results with reference to study objectives | Pages 15 - 18 |
| Limitations | 19 | Discuss limitations of the study, taking into account sources of potential or imprecision. Discuss both direction and magnitude of any potential bias | Pages 21 & 22 |
| Interpretation | 20 | Give a cautious overall interpretation of results considering objectives, limitations, multiplicity of analyses, results from similar studies, and other relevant evidence | Page 22 |
| Generalisability | 21 | Discuss the generalisability (external validity) of the study results | Page 22 |
| **Other Information** | | | |
| Funding | 22 | Give the source of funding and the role of the funders for the present study and, if applicable, for the original study on which the present article is based | Page 23 |
| Accessibility of protocol, raw data, and programming code |  | .. |  |

**Table 2 Sample characteristics (*n*=33,420)**

| **Characteristics** | **%** |
| --- | --- |
|  |  |
|  |  |
| Age, Mean (SD) | 10.6 (3.7) |
| Age Groupings, % (in years) |  |
| 5-8 | 33.9 |
| 9-12 | 32.7 |
| 13-17 | 33.4 |
| Male, % | 50.7 |
| **Population Group** |  |
| White | 65.1 |
| Asian | 21.3 |
| Black | 5.4 |
| Latin American | 1.2 |
| Indigenous | 5.0 |
| Other & Multiple | 2.0 |
|  |  |
| Low-income household, % | 20.0 |
|  |  |
| **Family Structure, %** |  |
| One and no biological parent in the home | 28.4 |
| Two biological parents in the home | 71.6 |
| **Parental Education, %** |  |
| High school and less | 20.9 |
| Less than bachelor degree | 39.4 |
| Bachelor’s degree and above | 39.7 |
|  | |

**Table 3: Rotated Factor Loadings from Exploratory Factor Analysis Models of CFM Questions**

| **CFM question** | **Factor 1** | **Factor 2** | **Uniqueness** |
| --- | --- | --- | --- |
|  |  |  |  |
| Selfcare | **0.7622** | 0.0133 | **0.3677** |
| Communication | **0.7649** | -0.1155 | **0.4032** |
| Learning | **0.8395** | -0.0824 | **0.2686** |
| Remembering | **0.7634** | -0.0510 | **0.3795** |
| Concentrating | **0.6783** | 0.0828 | **0.4273** |
| Accepting Change | **0.5184** | 0.3191 | **0.4376** |
| Behaviour | **0.5793** | 0.2289 | **0.434** |
| Relationships | **0.4901** | 0.3162 | **0.4724** |
| Anxiety | 0.0410 | **0.8863** | **0.1856** |
| Depression | -0.0040 | **0.7909** | **0.3411** |
| **Note:** Bolded items represent highest factor loadings. | | | |


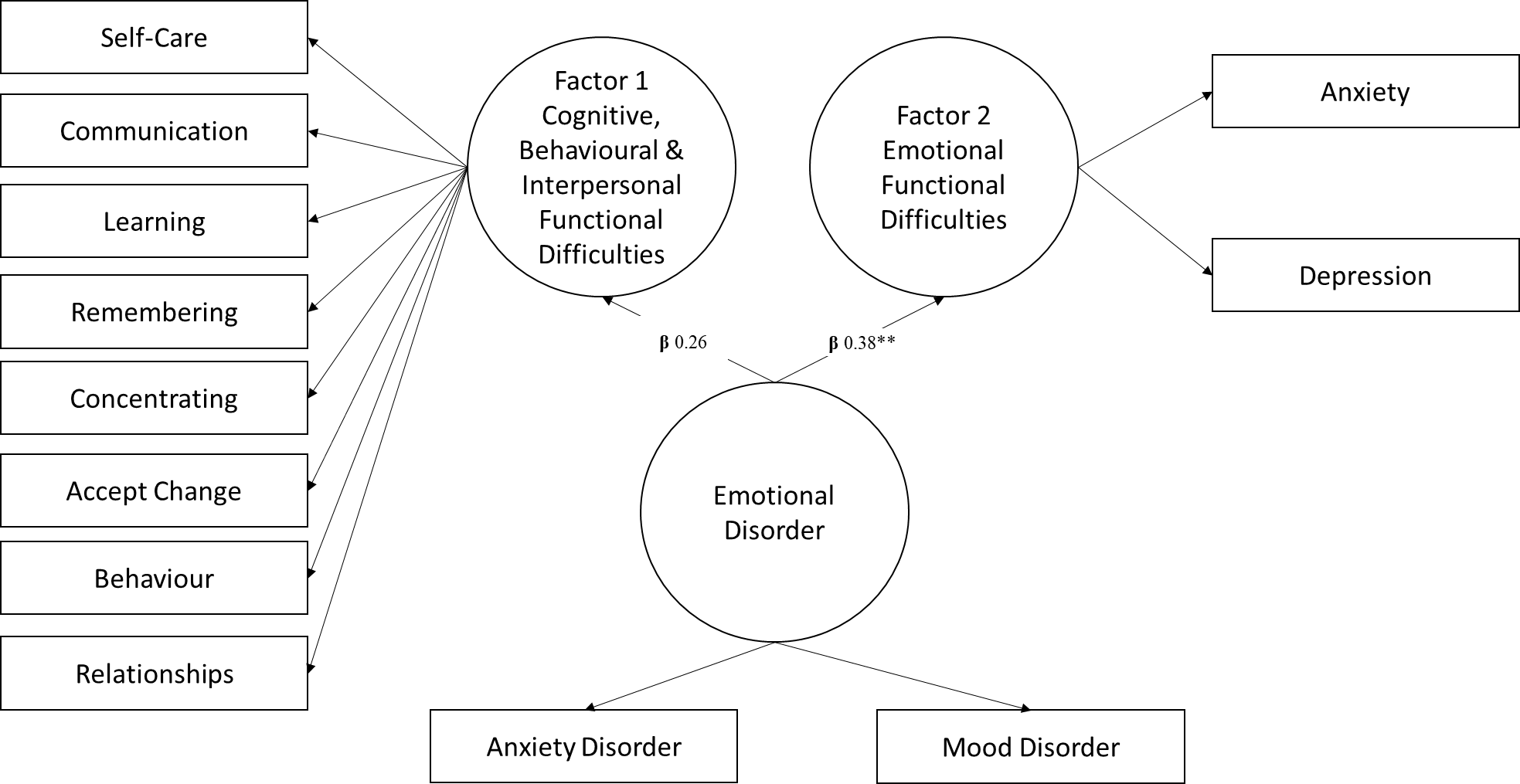


**Figure 1: An example of structural equational model for external validity using Long Term Health Condition of anxiety and mood disorders diagnosed by a health professional.**

Anxiety and mood disorder were measured as observed variables (coded as present = 1)**,** this was then regressed onto the CFM latent factors. Results indicated that the emotional disorders of anxiety and depression were more strongly associated with Factor 1 (the emotional functional difficulty factor) (**β=0.38)**, compared to Factor 3 (the cognitive and interpersonal functional difficulty factor) (**β=0.26)**. The difference in these coefficients was examined using Wald Chi-square test that indicated a significant difference of - 0.12 (p<.001). This SEM was repeated for all the instrumental variables. All SEM models met model fit criteria.
